# Antifungal susceptibility testing and determination of local epidemiological cut-off values for *Candida* species isolated from women with vulvovaginal candidiasis

**DOI:** 10.1128/spectrum.02488-24

**Published:** 2025-01-23

**Authors:** Vasiliki Kroustali, Lamprini Kanioura, Esmeralda Resoulai, Maria Siopi, Stavroula Antonopoulou, Joseph Meletiadis

**Affiliations:** 1 Clinical Microbiology Laboratory, “Attikon” University General Hospital, Medical School, National and Kapodistrian University of Athens, Athens, Greece; 2 Molecular Microbiology and Immunology Laboratory, Department of Biomedical Sciences, University of West Attica, Athens, Greece; 3 “MycoLab”, Diagnostic Laboratory of Sexually Transmitted Diseases, Specific Infectious Diseases, Fungal, Microbiological and Cytologic Examinations, Athens, Greece; 4 Department of Clinical Microbiology, General Hospital "G. Gennimatas", Athens, Greece; University of Debrecen, Debrecen, Hungary

**Keywords:** vulvovaginal candidiasis, antifungal susceptibility testing, local epidemiological cut-off values, resistance

## Abstract

The lack of clinical breakpoints and epidemiological cut-off values (ECOFFs) for antifungals prescribed for vulvovaginal candidiasis (VVC) make interpretation of antifungal susceptibility data difficult. This leads to empirical prescribing, poor clinical management and emergence of resistance. The *in vitro* susceptibilities of 152 *Candida albicans*, 105 *Candida parapsilosis*, 31 *Nakaseomyces glabratus,* and 8 *Pichia kudriavzevii* VVC isolates against eight antifungals, were determined according to the European Committee on Antimicrobial Susceptibility Testing (EUCAST) E.Def 7.4. The minimum inhibitory concentration (MIC) distributions were analyzed and local ECOFFs were determined visually and statistically. The *in vitro* activity of azoles was correlated with fluconazole susceptibility and clinical data were evaluated. The MICs of various azoles showed a significant correlation with the MICs of fluconazole and fluconazole non-wild type (WT) isolates had significantly higher MICs for other azoles. The estimated local ECOFFs for *C. albicans* were 0.016 mg/L (ketoconazole, clotrimazole), 0.06 mg/L (miconazole, econazole, itraconazole), 1 mg/L (fenticonazole), and 3,200 mg/L (boric acid). For *C. parapsilosis*, local ECOFFs were 0.06 mg/L (ketoconazole, clotrimazole, itraconazole), 1 mg/L (miconazole, econazole), 2 mg/L (fenticonazole), and 3,200 mg/L (boric acid). For *N. glabratus*, they were 1 mg/L (ketoconazole, clotrimazole, miconazole, itraconazole), 2 mg/L (econazole, fenticonazole), and 12,800 mg/L (boric acid). Non-WT isolates were detected for azoles in *N. glabratus* (10%–35%), *C. albicans* (5%–16%), and *C. parapsilosis* (≤ 3%). All isolates were WT for boric acid. Local ECOFFs were established for three major *Candida* species causing VVC, guiding the identification of non-WT isolates potentially associated with treatment failure.

**IMPORTANCE:**

The interpretation of *in vitro* susceptibility data of *Candida* isolates from women with vulvovaginal candidiasis (VVC) is challenging due to the lack of clinical breakpoints (CBPs) and epidemiological cut-off values (ECOFFs) for drugs used in VVC. In the present study, local ECOFFs were established for three major *Candida* species causing VVC, guiding the identification of non-wild type isolates potentially associated with treatment failure. This paper provides the framework for developing ECOFFs and ultimately CBPs that would help guide antifungal therapy of VVC.

## INTRODUCTION

Vulvovaginal candidiasis (VVC) is considered the most prevalent *Candida* infection in humans (1). It is estimated that approximately 20%–30% of asymptomatic women worldwide are colonized by *Candida* species (2) and around 75% of women will have at least one episode of symptomatic VVC in their lifetime (3). Despite prevailing evidence suggesting that *Candida albicans* is the predominant etiological agent, non-*albicans Candida* (NAC) species are increasingly implicated in VVC (4). Recent studies have reported alarming prevalence rates for *Nakaseomyces glabratus* (formerly known as *Candida glabrata,* 3.4%–36.7%), *Pichia kudriavzevii*, (formerly known as *Candida krusei,* 0.96%–17.2%), *Candida parapsilosis* (0.96%–45.1%), and *Candida tropicalis* (0.96%–11.1%) (4). The American Centers for Disease Control and Prevention have acknowledged that these species may not respond as anticipated to a treatment designed to manage uncomplicated VVC and that 10%–20% of women will have complicated VVC, requiring advanced diagnostic and therapeutic attention (5).

*In vitro* antifungal susceptibility testing (AFST) is necessary for therapeutic purposes to choose the most effective drug as well as for epidemiological purposes to monitor antifungal resistance (6). Epidemiological trends in VVC indicate that AFST is more crucial than ever, given not only the rising prevalence of NAC but also the increasing resistance of *C. albicans* to azoles contributing to high rates of disease recurrence (7). Azole-resistant vaginitis—often resulting from frequent and prolonged exposure to a specific agent—poses a significant challenge to successful treatment, primarily due to the limited availability of alternative systemic and local agents (8). The European Committee on Antimicrobial Susceptibility Testing (EUCAST) has developed the standard protocol E. Def 7.4 for minimum inhibitory concentrations (MIC) determination of yeasts (9) and has defined clinical breakpoints (CBPs) for MIC interpretation. As there are no CBPs for all drugs, epidemiological cut-off values (ECOFFs) have been determined based on aggregated MIC data (6). ECOFFs can be used to determine non-wild type (WT) populations for a given drug and species with acquired resistance that likely will not respond to antifungal therapy.

So far, there are no CBPs or ECOFFs for the majority of antifungals administered to women with VVC, making its therapeutic approach primarily empirical (10). Available treatment for acute VVC includes the oral triazoles fluconazole, itraconazole, and ketoconazole or antifungal agents applied locally like the imidazoles clotrimazole, econazole, fenticonazole, miconazole, the polyenes nystatin, ciclopiroxolamine, and boric acid (2, 5). For recurrent vulvovaginal candidiasis (RVVC) cases, dose‐reducing suppression therapy with fluconazole or itraconazole is recommended (2). Among the drugs used for VVC, EUCAST has established CBPs and/or ECOFFs for *C. albicans, N. glabratus, C. parapsilosis,* and *P. kudriavzevii,* for fluconazole and itraconazole derived from clinical data from invasive and oesophageal candidiasis (11). Notably, 20%–50% of women treated with fluconazole or itraconazole report temporary symptom relief (12), underscoring the need to establish resistance criteria for alternative treatments. As VVC and oesophageal candidiasis are both mucosal infections, the CBPs and ECOFFs of fluconazole and itraconazole that are used for oesophageal candidiasis may be valid for VVC, although there are differences between the two anatomical sites (e.g. pH). EUCAST has acknowledged that ECOFFs of topical agents or agents that reach the site of infection at high concentrations may underestimate the activity of some agents in topical preparations (13).

Based on these grounds, we assessed the *in vitro* susceptibility profile of different *Candida* species to eight topical and systemically applied antifungal drugs commonly used for the treatment of VVC with the EUCAST E.Def 7.4 method (9) and analyzed the MIC distributions in order to determine local ECOFFs based on different statistical approaches, cross-resistance to fluconazole and correlation with the clinical outcome.

## MATERIALS AND METHODS

### Patients and isolates

During 2019–2021, women from various regions in Greece visited the private diagnostic laboratory “MycoLab” (Athens, Greece) with symptoms suggestive of VVC. High vaginal samples were collected from which *Candida* strains were isolated leading to a VVC diagnosis. A total of 152 *C*. *albicans*, 105 *C*. *parapsilosis sensu stricto* (SS), 31 *N*. *glabratus,* and 8 *P*. *kudriavzevii* clinical isolates were tested, all recovered from adult women (aged 20–69 years) with VVC. Among these patients, 18% had been exposed to antifungals prescribed for VVC within the past month (7.5% had received fluconazole, 1% itraconazole, 3% a combination of itraconazole and fluconazole, and 6.5% topical azoles). All women were asked to provide written consent, and the study database was anonymized to ensure that individuals could not be identified. Species identification was performed by matrix-assisted laser desorption ionization-time of flight mass spectrometry (Bruker Daltonics, Bremen, Germany). All isolates were stored at –70°C in normal sterile saline with 10% glycerol (AppliChem, Athens, Greece) until use. Before testing, they were revived by subculturing them onto Sabouraud glucose agar with gentamicin and chloramphenicol plates (SGC; Oxoid, Athens, Greece) at 35°C ± 2°C for 24 h. This medium is used in routine diagnostics to ensure purity. Preliminary studies showed that EUCAST MICs of common yeasts show no significant difference when subcultured on Sabouraud glucose agar with or without these antibiotics before testing.

### Antifungal susceptibility testing

Following species identification, all isolates were tested for susceptibility to azole antifungals. However, for boric acid, we randomly selected a subset of isolates from each species: 60 *C*. *albicans*, 56 *C*. *parapsilosis* SS, 21 *N*. *glabratus,* and 6 *P*. *kudriavzevii*. AFST was performed according to the EUCAST E.Def 7.4 protocol guidelines (9). The nutrient medium used was RPMI 1640 (with L-glutamine and without bicarbonate, buffered at pH 7.0 with MOPS) supplemented with glucose at a final concentration of 2%. Laboratory-grade pure powders of fluconazole, itraconazole, ketoconazole, econazole, clotrimazole (TCI, Athens, Greece), miconazole (Fluorochem, Athens, Greece), and fenticonazole (Sigma-Aldrich, Athens, Greece) were dissolved in dimethyl sulfoxide (Chem-Lab, Athens, Greece), while boric acid (TCI, Athens, Greece) was dissolved in sterile distilled water. Twofold serial final drug concentrations were prepared for fluconazole (0.06 to 64 mg/L), clotrimazole, econazole, miconazole, ketoconazole, itraconazole, and fenticonazole (0.004 to 2 mg/L), and boric acid (6.25 to 6,400 mg/L) following the ISO standard 20776-1 dilution method (14). The solutions were dispensed into 96-well microtitration plates with flat-bottom, tissue culture-treated wells (Nunc MicroWell; catalog no. 167008, Thermo Fisher Scientific, Athens, Greece). The plates were sealed in aluminum foil and stored frozen at –70°C until use. On the day of the experiment, they were thawed, inoculated with yeast suspensions (prepared in sterile distilled water and adjusted to the required concentration), and incubated at 35°C ± 2°C. The MICs were determined spectrophotometrically (OD at 530 nm) after 24 h as the lowest drug concentration with ≥50% fungal growth inhibition compared to the drug-free control (9).

Inoculum density check was performed on all isolates by spread plate counts on SGC agar plates. The recommended *C. krusei* ATCC 6258 and *C. parapsilosis* ATCC 22019 were used as quality control strains and were tested thrice.

### Data analysis

MIC distributions were constructed and the median and modal (range) MICs, geometric mean (GM) MICs, MIC_50_s, and MIC_90_s (the concentrations that inhibited 50% and 90%, respectively, of the isolates) were calculated for each drug and species. High off-scale MICs were converted to the next highest twofold concentration, while low off-scale MICs were left unchanged. The MIC data sets were logarithmically transformed (log_2_) to better approximate a normal distribution.

#### MIC correlation

In order to investigate cross-resistance between drugs, a correlation matrix was constructed to examine potential correlations between the MICs of antifungal agents tested for each *Candida* species. Pearson’s correlation coefficient (*r*) was calculated to determine the strength and direction of the connection between the variables. A value of *r* nearing +1 implies a robust positive correlation, while one approaching −1 indicates a negative correlation. Conversely, a value of *r* close to 0 signifies minimal correlation. Because of multiple comparisons, a *P* value of < 0.001 was considered significant. The hypothesis is that the MICs of drugs that share resistance mechanisms will be significantly correlated, and therefore a WT strain to one drug (i.e., not harboring acquired resistance mechanisms) is very likely to be WT to correlated drugs.

#### Evaluation based on fluconazole susceptibility

We further investigated any statistically significant difference in the MICs of other drugs between fluconazole-WT and -non-WT strains with a one-way analysis of variance (ANOVA) followed by the Holm-Sidak multiple comparison post hoc test. A *P* value of < 0.05 indicated a significant difference. Furthermore, the receiver operating characteristic (ROC) analysis was used to determine the optimal MIC cut-off value (the MIC with the highest likelihood ratio [LR]) that separates fluconazole WT from non-WT isolates for each drug. These MIC cut-off values are likely to be close to the ECOFFs if fluconazole MICs are correlated with the MICs of other drugs. The sensitivity (Sen) and specificity (Spe) of the MIC cut-off value to detect azole resistance were also calculated. Finally, a similar analysis was made for fluconazole-susceptible (both susceptible and susceptible, increased exposure) and fluconazole-resistant isolates. The hypothesis for this comparison is the following: as fluconazole CBPs were determined based also on clinical data from oesophageal candidiasis, they may apply to other mucosal infections like VVC. Hence a fluconazole-susceptible or -resistant isolate may be susceptible or resistant to other azoles, respectively. The susceptible, increased exposure category, which indicates that a strain may be treatable with higher fluconazole doses, may also be applicable to other azoles providing a higher dose can be given or the drug reaches a high concentration in the vaginal fluid.

#### Local ECOFF determinations

ECOFFs are defined as the highest MIC value of the WT distribution. Local ECOFFs were determined by visual inspection of the MICs histograms (the eyeball method) (15), statistically using the ECOFFinder program (available at https://www.eucast.org/mic_and_zone_distributions_and_ecoffs) with the selected confidence levels ranging from 97.5% to 99.5% of the modeled distribution, and with the derivatization method (dECOFFs) by calculating the numerical second derivative at each MIC of the MIC distribution (16). The consensus local ECOFF was then determined as the common ECOFF among most methods. ECOFFs were estimated for species with 15 or more isolates; thus, they were not determined for *P. kudriavzevii*.

All data were analyzed using the statistics software package GraphPad Prism, version 10.0, for Windows (GraphPad Software, San Diego, CA, USA).

### Clinical data

In order to assess the clinical efficacy of antifungal drugs for VVC, we reviewed the literature and summarized all clinical studies on antifungal therapy against VVC where outcome per species was reported. For drugs with limited clinical trials, studies without species-specific outcome data was considered relevant to *C. albicans* due to the high prevalence of this species in the disease. In addition, we retrieved clinical data of the isolates used in the present study for patients who were treated with fluconazole or itraconazole where there was information on dose, clinical outcome, and the MIC of the pathogen. Response to therapy was defined as both mycological (negative microscopy/culture) and clinical (symptom resolution) cure. Otherwise, cases were deemed failures.

## RESULTS

### MIC data of quality control strains

The MIC data resulting from testing the quality control strains *C. parapsilosis* ATCC 22019 and *C. krusei* ATCC 6258, in three different batches of microtiter plates, are presented in Table 1. The results were within EUCAST defined acceptable range for fluconazole and itraconazole (11) and were consistent with EUCAST or the Clinical and Laboratory Standards Institute (CLSI) data sourced from the literature for ketoconazole (17), miconazole (18), clotrimazole (18), fenticonazole (19), and boric acid (20). There were no available data for econazole.

**TABLE 1:** MIC data for quality control strains *C. parapsilosis* ATCC 22019 and *C. krusei* ATCC 6258^*c*^

Drug	Quality control strain	Replicate 1 MIC (mg/L)	Replicate 2 MIC (mg/L)	Replicate 3 MIC (mg/L)	Target (range) MIC (mg/L)	Method, Reference
FLZ	*C. parapsilosis* ATCC 22019	1	1	1	1 (0.5–2)	EUCAST (11)
	*C. krusei* ATCC 6258	16	16	32	32 (16–64)	EUCAST (11)
KTZ	*C. parapsilosis* ATCC 22019	0.03	0.016	0.03	0.03 (0.01–0.06)^*a*^	EUCAST (17)
	*C. krusei* ATCC 6258	0.25	0.125	0.25	0.125 (0.06–0.25)^*a*^	EUCAST (17)
MCZ	*C. parapsilosis* ATCC 22019	0.5	0.5	0.5	0.5 (0.25–0.5)^*a*^	EUCAST (18)
	*C. krusei* ATCC 6258	0.5	1	1	1 (0.25–2)^*a*^	EUCAST (18)
ECZ	*C. parapsilosis* ATCC 22019	0.5	0.5	1	NA	NA
	*C. krusei* ATCC 6258	0.5	1	0.5	NA	NA
ITZ	*C. parapsilosis* ATCC 22019	0.03	0.06	0.06	0.06 (0.03–0.125)	EUCAST (11)
	*C. krusei* ATCC 6258	0.03	0.06	0.125	0.06 (0.03–0.125)	EUCAST (11)
CLZ	*C. parapsilosis* ATCC 22019	0.06	0.03	0.06	0.06 (0.03–0.06)^*a*^	EUCAST (18)
	*C. krusei* ATCC 6258	0.03	0.06	0.06	0.06 (0.016–0.06)^*a*^	EUCAST (18)
FNZ	*C. parapsilosis* ATCC 22019	2	2	1	0.25 (0.03–0.25)^*a*^	EUCAST (19)
	*C. krusei* ATCC 6258	2	2	2	0.5 (0.06–2)^*a*^	EUCAST (19)
BA	*C. parapsilosis* ATCC 22019	1,600	1,600	1,600	1,930^*b*^	CLSI (20)
	*C. krusei* ATCC 6258	400	800	800	970^*b*^	CLSI (20)

^*a*^ MIC range of the control stain according to other studies. Target MIC was chosen as the modal MIC among the trials of the study or the median of the suggested range if there was no MIC distribution available.

^*b*^ Range not available.

^*c*^ BA, boric acid; CLSI, Clinical and Laboratory Standards Institute; CLZ, clotrimazole; ECZ, econazole; EUCAST, European Committee on Antimicrobial Susceptibility Testing; FLZ, fluconazole; FNZ, fenticonazole; KTZ, ketoconazole; ITZ, itraconazole; MCZ, miconazole; MIC, minimum inhibitory concentration; NA, not available.

### MIC distributions of clinical isolates

The EUCAST MICs for the eight antifungal compounds against *Candida* species are displayed in Table 2. Wider MIC distributions were found for *C. albicans* compared to other *Candida* species. *C. albicans* exhibited the lowest GM MIC values for all azoles, compared to NAC species. The lowest GM MIC was found for clotrimazole against *C. albicans* (0.010 mg/L), itraconazole against *C. parapsilosis* SS (0.021 mg/L) and *P. kudriavzevii* (0.074 mg/L), and econazole against *N. glabratus* (0.208 mg/L). The GM MIC values of boric acid were in ascending order: *P. kudriavzevii* (800 mg/L) < *C. parapsilosis* SS (1,160 mg/L) < *C. albicans* (1,459 mg/L) < *N. glabratus* (2,691 mg/L). Based on the EUCAST CBPs for fluconazole and itraconazole, resistance was found for 6/152 (4%) and 8/152 (5%) *C*. *albicans* isolates, respectively, none of the *C. parapsilosis* SS isolates and 7/31 (23%) *N*. *glabratus* isolates to fluconazole.

**TABLE 2:** MIC distributions of antifungal drugs used for the treatment of VVC^*b*^

Species (no. of isolates)	Drug^*a*^	0.004	0.008	0.016	0.03	0.06	0.125	0.25	0.5	1	2	4	8	16	32	64	>64	200	400	800	1,600	3,200	6,400	GM MIC	MIC_50_	MIC_90_	% Non-WT
*C. albicans* (152 for azoles, 60 for BA)	FLZ^ot^					5	45	**75**	3	*8*	*7*	*3*	*4*	*2*										0.294	0.25	2	16
	KTZ^ot^	16	**100**	11		*4*	*1*	*9*	*7*	*1*		*3*												0.014	0.008	0.25	16
	MCZ^t^	7	26	**52**	42	1	*1*	*6*	*9*		*8*													0.029	0.016	0.5	16
	ECZ^t^		22	**57**	41	9	*3*	*8*	*5*	*6*	*1*													0.030	0.016	0.25	15
	ITZ^o^	11	49	**58**	23	3	*8*																	0.014	0.016	0.03	5
	CLZ^t^	14	**81**	45	*8*	*3*	*1*																	0.010	0.008	0.016	8
	FNZ^t^						16	53	**65**	11	*3*	*4*												0.387	0.5	1	5
	ΒΑ^t^																		2	5	**52**	1		1,459	1,600	1,600	0
*C. parapsilosis* SS (105 for azoles, 56 for BA)	FLZ^ot^							1	32	**72**														0.799	1	1	0
	KTZ^ot^			6	**90**	8	*1*																	0.032	0.03	0.03	1
	MCZ^t^						12	**47**	46															0.313	0.25	0.5	0
	ECZ^t^						3	23	**74**	4	*1*													0.430	0.5	0.5	1
	ITZ^o^		8	**60**	26	8	*3*																	0.021	0.016	0.06	3
	CLZ^t^			7	**80**	18																		0.033	0.03	0.06	0
	FNZ^t^								1	**77**	26	*1*												1.195	1	2	1
	ΒΑ^t^																	1	7	10	**37**	1		1,160	1,600	1,600	0
*N. glabratus* (31 for azoles, 21 for BA)	FLZ^ot^										1	7	**11**	5			*7*							13.68	8	>64	23
	KTZ^ot^				1		5	8	**10**		*1*	*6*												0.478	0.5	>2	23
	MCZ^t^				1	3	8	8	**10**	1														0.222	0.25	0.5	0
	ECZ^t^				2	4	**8**	**8**	4	5														0.208	0.25	1	0
	ITZ^o^						6	**10**	5	3	*1*	*6*												0.511	0.25	>2	23
	CLZ^t^					2	2	**8**	5	3	*7*	*4*												0.638	0.5	4	35
	FNZ^t^								8	**16**	4	*3*												1.046	1	2	10
	ΒΑ^t^																		1	2	5	**7**	6	2,691	3,200	6,400	0
*P. kudriavzevii* (8 for azoles, 6 for BA)	FLZ^ot^								|				1	1	**5**	1								26.91	32	64	ND
	KTZ^ot^			|			**3**	2	**3**															0.250	0.25	0.5	ND
	MCZ^t^					|	1	1	1		**5**													0.917	2	2	ND
	ECZ^t^					|			2	**5**	1													0.917	1	2	ND
	ITZ^o^				**3**	2|	1	2																0.074	0.06	0.25	ND
	CLZ^t^			|	1	**4**	2	1																0.080	0.06	0.25	ND
	FNZ^t^									2|	**6**													1.682	2	2	ND
	ΒΑ^t^																	1		**3**	2	|		800	800	1,600	ND

^*a*^ Topical (t) or oral (o) formulations available in Greece.

^*b*^ Drug concentrations, GM MICs, MIC_50_s, MIC_90_s are expressed in mg/L. Bold numbers indicate modal MICs, underlined numbers indicate off-scale MICs, italicized numbers represent non-WT isolates based on consensus local ECOFFs, and vertical lines show the local ECOFFs for *C. albicans* based on which most *P. kudriavzevii* isolates will be classified as non-WT in accordance to FLZ-WT status. BA, boric acid; CLZ, clotrimazole; ECZ, econazole; FNZ, fenticonazole; FLZ, fluconazole; GM, geometric mean; ITZ, itraconazole; KTZ, ketoconazole; MCZ, miconazole; ND, not determined; SS, *sensu stricto*.

### MIC correlation

A correlation matrix of the different drugs tested for each *Candida* species is shown in Table 3. Significant (P < 0.001) 01) correlation was found among the MICs of azoles for *C. albicans* (*r* > 0.40, except ketoconazole and fenticonazole) and *N. glabratus* (*r* > 0.64), but not for *C. parapsilosis* SS. For *C. albicans*, fluconazole MICs were moderately correlated with all other azoles (*r* 0.46–0.88), except ketoconazole (*r* 0.07) and fenticonazole (*r* 0.13), while for *N. glabratus* fluconazole MICs were highly correlated with the MICs of all other azoles (*r* 0.67–0.99). Boric acid MICs were not correlated with the MICs of azoles.

**TABLE 3:** Correlation matrices between MICs of all drugs for each species^*a*^^,^^*b*^

Species (No of isolates)	Drugs	FLZ	KTZ	MCZ	ECZ	ITZ	CLZ	FNZ	BA
*C. albicans* (152 for azoles, 60 for BA)	**FLZ**	1.000							
	**KTZ**	0.068	1.000						
	**MCZ**	**0.879**	0.061	1.000					
	**ECZ**	**0.705**	0.042	**0.697**	1.000				
	**ITZ**	**0.795**	0.127	**0.875**	**0.647**	1.000			
	**CLZ**	**0.464**	−0.018	**0.581**	**0.403**	**0.538**	1.000		
	**FNZ**	0.129	−0.008	0.128	0.117	0.127	0.144	1.000	
	**BA**	−0.292	−0.249	−0.158	−0.237	−0.191	−0.072	0.348	1.000
*C. parapsilosis* SS (105 for azoles, 56 for BA)	**FLZ**	1.000							
	**KTZ**	−0.048	1.000						
	**MCZ**	0.071	−0.097	1.000					
	**ECZ**	0.143	−0.037	0.205	1.000				
	**ITZ**	−0.085	−0.065	0.088	0.170	1.000			
	**CLZ**	−0.050	−0.005	0.042	0.289	0.111	1.000		
	**FNZ**	−0.036	−0.122	−0.045	−0.103	0.159	0.322	1.000	
	**BA**	−0.205	0.033	−0.092	0.100	0.038	−0.018	0.149	1.000
*N. glabratus* (31 for azoles, 21 for BA)	**FLZ**	1.000							
	**KTZ**	**0.991**	1.000						
	**MCZ**	**0.666**	**0.637**	1.000					
	**ECZ**	**0.903**	**0.892**	**0.719**	1.000				
	**ITZ**	**0.964**	**0.965**	**0.649**	**0.923**	1.000			
	**CLZ**	**0.844**	**0.827**	**0.692**	**0.737**	**0.778**	1.000		
	**FNZ**	**0.853**	**0.855**	**0.661**	**0.862**	**0.872**	**0.759**	1.000	
	**BA**	0.059	0.498	0.118	0.235	0.010	0.085	−0.108	1.000

^*a*^ Bold numbers correspond to *P* values < 0.0001.

^*b*^ BA, boric acid; CLZ, clotrimazole; ECZ, econazole; FNZ, fenticonazole; FLZ, fluconazole; ITZ, itraconazole; KTZ, ketoconazole; MCZ, miconazole; SS, *sensu stricto*.

### Comparison of azoles MICs between fluconazole-WT and -non-WT isolates

The median (range) azoles MICs for the fluconazole-WT and -non-WT *C. albicans* isolates are presented in Table 4. Overall, fluconazole-non-WT strains exhibited significantly higher azole MIC values compared to those of fluconazole-WT (*P* < 0.001 for all azoles), except fenticonazole (*P* = 0.110). No significant differences were observed for boric acid MICs (data not shown). ROC analysis showed that the optimal MIC cut-offs were 0.5 mg/L for fluconazole (LR = 43, Sen = 100%, Spe = 98%), 0.125 mg/L for miconazole (LR = 117, Sen = 92%, Spe = 99%), and econazole (LR = 101, Sen = 79%, Spe = 99%), and 0.016 mg/L for itraconazole (LR = 9, Sen = 88%, Spe = 90%), clotrimazole (LR = 27, Sen = 42%, Spe = 98%), and ketoconazole (LR = 39, Sen = 92%, Spe = 98%). For fenticonazole and boric acid the LRs were small (< 4). For *N. glabratus*, results are shown in the next section as the ECOFF is the same as the CBP for the susceptible, increased exposure category (16 mg/L), and thus, WT/non-WT status corresponds to susceptible, increased exposure/resistant classification.

**TABLE 4:** Median (range) MICs (mg/L) of fluconazole-resistant and -susceptible *Candida* isolates to other azoles and boric acid used in the treatment of VVC^*b*^

Species (no. of isolates)	FLZ	KTZ	MCZ	ECZ	ITZ	CLZ	FNZ
*C. albicans* FLZ-non-WT MICs (24)	2 (1–16)	0.25 (0.008–1)	0.5 (≤0.004–2)	0.25 (0.016–1)	0.03 (0.016–0.125)	0.016 (0.008–0.125)	0.5 (0.125–2)
*C. albicans* FLZ-WT MICs (128)	0.25 (≤0.06–0.5)	0.008 (≤0.004–>2)	0.016 (≤0.004–0.5)	0.016 (0.008–2)	0.016 (≤0.004–0.03)	0.008 (≤0.004–0.03)	0.25 (0.125–>2)
ANOVA *P* value*^a^*	<0.0001	<0.0001	<0.0001	<0.0001	<0.0001	<0.0001	0.110
MIC cut-off for FLZ–WT	0.5	0.016	0.125	0.125	0.016	0.016	0.5
AUC of ROC curve	1	0.96	0.92	0.95	0.94	0.78	0.69
*P* value	<0.0001	<0.0001	<0.0001	<0.0001	<0.0001	<0.0001	0.003
*C. albicans* FLZ-R MICs (6)	8 (8–16)	0.5 (0.5–1)	2 (2)	1 (0.5–1)	0.125 (0.06–0.125)	0.03 (0.016–0.06)	1 (0.25–2)
*C. albicans* FLZ-S MICs (146)	0.25 (≤0.06–4)	0.008 (≤0.004–>2)	0.016 (≤0.004–2)	0.016 (0.008–2)	0.016 (≤0.004–0.125)	0.008 (≤0.004–0.125)	0.5 (0.125–>2)
ANOVA *P* value	<0.0001	<0.0001	<0.0001	<0.0001	<0.0001	0.037	0.606
MIC cut-off for FLZ-S	4	0.125	1	0.5	0.06	0.016	0.5
AUC of ROC curve	1	0.97	0.99	0.97	0.99	0.94	0.75
*P* value	<0.0001	<0.0001	<0.0001	<0.0001	<0.0001	<0.0001	0.042
*N. glabratus* FLZ-R/non-WT MICs (7)	>64 (>64–>64)	>2 (>2–>2)	0.5 (0.25–1)	1 (0.5–1)	>2 (2–>2)	4 (0.5–>2)	2 (1–>2)
*N. glabratus* FLZ-S/WT MICs (24)	8 (2–16)	0.25 (0.03–0.5)	0.25 (0.03–0.5)	0.125 (0.03–0.5)	0.25 (0.125–1)	0.5 (0.06–2)	1 (0.5–1)
ANOVA *P* value	<0.0001	<0.0001	0.003	<0.0001	<0.0001	<0.0001	<0.0001
MIC cut-off for FLZ-S	16	0.5	0.5	0.5	1	1	1
AUC of ROC curve	1	1	0.87	0.99	1	0.96	1
*P* value	<0.0001	<0.0001	0.003	0.0001	<0.0001	<0.0001	<0.0001
*P. kudriavzevii* FLZ-R MICs (8)	32 (8–64)	0.5 (0.125–0.5)	2 (0.125–2)	1 (0.5–2)	0.03 (0.03–0.25)	0.06 (0.03–0.25)	2 (1–2)

^*a*^ Obtained from the Holm-Sidak multiple comparison post-test for comparing the MICs of each drug between FLZ-R and FLZ-S isolates, a *P* value < 0.05 was considered significant.

^*b*^ AUC, area under the curve; CLZ, clotrimazole; ECZ, econazole; FNZ, fenticonazole; FLZ, fluconazole; ITZ, itraconazole; KTZ, ketoconazole; MCZ, miconazole; R, resistant; S, susceptible.

### Comparison of azoles MICs between fluconazole-susceptible and -resistant isolates

The median (range) MICs of fluconazole-susceptible and -resistant *C. albicans* and *N. glabratus* strains are presented in Table 4, data for *P. kudriavzevii* are also displayed. For *C. albicans*, fluconazole-resistant strains exhibited significantly higher MIC values compared to those of fluconazole-susceptible for all azoles (*P* ≤ 0.037), except fenticonazole (*P* = 0.606). Regarding *N. glabratus*, fluconazole-resistant strains exhibited significantly higher MICs for all azoles compared to fluconazole-susceptible strains (*P* ≤ 0.003). For both species, no significant differences were observed for boric acid MICs (data not shown). The optimal MIC cut-offs based on the ROC analysis for *C. albicans* were 4 mg/L for fluconazole (LR > 100, Sen = 100%, Spe = 100%), 0.125 mg/L for ketoconazole (LR = 29, Sen = 100%, Spe = 97%), 1 mg/L for miconazole (LR = 73, Sen = 100%, Spe = 99%), 0.5 mg/L for econazole (LR = 61, Sen = 83%, Spe = 99%), 0.06 mg/L for itraconazole (LR = 41, Sen = 83%, Spe = 98%), 0.016 mg/L for clotrimazole (LR = 17, Sen = 83%, Spe = 95%). For fenticonazole and boric acid the LRs were small (< 5). The corresponding MIC cut-offs for *N. glabratus* were 16 mg/L for fluconazole (LR > 100, Sen = 100%, Spe = 100%), 0.5 mg/L for ketoconazole (LR > 100, Sen = 100%, Spe = 100%), miconazole (LR = 4, Sen = 86%, Spe = 79%), and econazole (LR = 12, Sen = 100%, Spe = 92%) and 1 mg/L for clotrimazole (LR = 6, Sen = 100%, Spe = 83%), fenticonazole (LR > 100, Sen = 100%, Spe = 100%), and itraconazole (LR > 100, Sen = 100%, Spe = 100%). For boric acid, the LR was small (< 4).

### Local ECOFFs

The estimated local ECOFFs, using three different methods, for each *Candida* species are shown in Table 5. Out of the 24 drug-species pairs (three species, eight drugs), the dECOFFs could not be estimated for *N. glabratus* for clotrimazole and boric acid. Regarding the remaining 22 drug-species pairs, the values of the statistical 99% or 99.5% ECOFF, those obtained by the eyeball method, and the dECOFFs were the same in 18 (82%) and 1 twofold dilution different in 4 (18%, itraconazole and clotrimazole for *C. albicans*, fluconazole and miconazole for *N. glabratus*). For the latter, the consensus local ECOFFs were determined based on the eyeball method. Regarding *N. glabratus* and clotrimazole, there was significant overlapping between the MICs of WT and non-WT isolates, as the consensus ECOFF (1 mg/L) falls between the two modal MICs (0.25 mg/L and 2 mg/L) of the distribution, also shown in Table 2. The MIC cut-offs associated with fluconazole susceptibility for *C. albicans* were the same as the corresponding local ECOFFs except for ketoconazole, miconazole, and econazole which were higher. For *N. glabratus*, MIC cut-offs associated with fluconazole susceptibility were 1 twofold dilution lower than the corresponding local ECOFFs except for itraconazole and clotrimazole which were the same.

**TABLE 5:** Epidemiological cut-off values (mg/L) for azole drugs and boric acid^*b*^

Species (no. of isolates)	Drug	ECOFF 95%	ECOFF 97.5%	ECOFF 99%	ECOFF 99.5%	Eyeball method	dECOFF	MIC cut-off of FLZ-WT	Consensus ECOFF	MIC cut-off of FLZ-S
*C. albicans* (152 for azoles, 60 for BA)	FLZ	0.25	0.25	0.5	0.5	0.5	0.5	0.5	0.5	4
	KTZ	0.016	0.016	0.016	0.016	0.016	0.016	0.016	0.016	0.125
	MCZ	0.06	0.06	0.06	0.06	0.06	0.06	0.125	0.06	1
	ECZ	0.06	0.06	0.06	0.06	0.06	0.06	0.125	0.06	0.5
	ITZ	0.03	0.03	0.03	0.06	0.06	0.03	0.016	0.06	0.06
	CLZ	0.016	0.016	0.03	0.03	0.016	0.03	0.016	0.016	0.016
	FNZ	1	1	1	2	1	1	ND	1	ND
	BA	1,600	1,600	3,200	3,200	3,200	3,200	ND	3,200	ND
*C. parapsilosis* SS (105 for azoles, 56 for BA)	FLZ	1	1	2	2	2	2	ND	2	ND
	KTZ	0.06	0.06	0.06	0.06	0.06	0.06	ND	0.06	ND
	MCZ	1	1	1	1	1	1	ND	1	ND
	ECZ	0.5	1	1	1	1	1	ND	1	ND
	ITZ	0.03	0.03	0.03	0.06	0.06	0.06	ND	0.06	ND
	CLZ	0.06	0.06	0.06	0.06	0.06	0.06	ND	0.06	ND
	FNZ	2	2	2	2	2	2	ND	2	ND
	BA	3,200	3,200	3,200	3,200	3,200	3,200	ND	3,200	ND
*N. glabratus* (31 for azoles, 21 for BA)	FLZ	16	16	32	32	16	16	16	16	16
	KTZ	1	1	1	2	1	1	0.5	1	0.5
	MCZ	1	2	2	2	1	1	0.5	1	0.5
	ECZ	1	2	2	4	2	2	1	2	1
	ITZ	0.5	0.5	1	1	1	1	1	1	1
	CLZ	1	2	2	2	1	ND	1	1	1
	FNZ	2	2	2	2	2	2	ND	2	ND
	BA^*a*^	6,400	6,400	12,800	12,800	12,800	ND	ND	12,800	ND

^*a*^ Extrapolated.

^*b*^ BA, boric acid; CLZ, clotrimazole; ECZ, econazole; FNZ, fenticonazole; FLZ, fluconazole; ITZ, itraconazole; KTZ, ketoconazole; MCZ, miconazole; ND, not determined; SS, *sensu stricto*.

### Clinical data

#### Review

Clinical studies are summarized in Table 6. Mycological cure rates for *C. albicans* VVC were 58%–97% with a single dose of 150 mg fluconazole per os (PO), 53%–87% with 150–200 mg fluconazole (PO) every 4 days for three doses, 50%–94% with 200 mg itraconazole (PO) or 100 mg clotrimazole (intravaginally) daily for 3 days, 66.5%–93% with a single dose of 500 mg clotrimazole (intravaginally), 67%–80% with 400 mg ketoconazole (PO) for 5 days or 200 mg ketoconazole (PO) for 6 days, 86%–97.5% with 400 mg miconazole (intravaginally) for 3–6 days, 76%–81% with 100 mg miconazole daily for 14 days, 64%–80% with either a 1,200 mg single dose of miconazole or 1,200 mg for days 1 and 4, 80%–84.8% with 150 mg econazole (intravaginally) daily for 3 days, 73.3%–97.5% with a single dose of 600 mg fenticonazole (intravaginally) or 200 mg fenticonazole daily for 3 days or 5 g of 2% fenticonazole cream applied daily for 7 days or a 1,000 mg single dose, 72%–92% (except one study that reported 61% mycological cure rate among women with VVC and diabetes), with 600 or 300 mg boric acid (intravaginally) daily for 14 days and 95%–100% with 600 mg boric acid twice a day.

**TABLE 6:** Summary of clinical trials with species-specific outcome data

Reference	Trial	Number of patients	Type of patients	Species	Drugs and doses	Outcome	Comments
Ray et al. (21)	Open-label randomized controlled trial	100	Acute VVC and diabetes	*N. glabratus* SC (61%)	150 mg PO single dose of fluconazole (*n* = 55)	*N. glabratus* SC: mycological cure rates 63.6% (BA) and 28.6% (fluconazole)	For *N. glabrat*us SC BA demonstrated a significantly higher cure rate of 72% compared to 33% for fluconazole (*P* = 0.01)
				*C. albicans* (29%)	600 mg BA daily intravaginally for 14 days (*n* = 56)	*C. albicans*: mycological cure rates 61% (BA) and 86% (fluconazole)	
				Other (10%)			
Swate and Weed (22)	Open-label prospective study	40	Acute VVC/RVVC	*C. albicans* (93%)	600 mg BA 2 /day intravaginally for 14 days	14 days follow-up: 100% mycological cure rate	
				Other (7%)		30 days follow-up: 95% mycological cure rate	
Nyirjesy et al. (23)	Observational, prospective study	74	RVVC/chronic VVC	*C. albicans* (68%)	200 mg fluconazole PO every 4th day for three doses (*n* = 59)	C*. albicans*: mycological cure rate 100% (fluconazole)	
				*N. glabrat*us SC (16%)	200 mg itraconazole PO daily for 14 days (*n* = 6)	NAC spp.: mycological cure rate 25% (fluconazole)	
				*C. parapsilosis* SC (5%)	100 mg clotrimazole daily intravaginally for 14 days (*n* = 7)	NAC spp.: mycological cure rates 85% (boric acid), 57% (clotrimazole) and 50% (itraconazole)	
				Other (11%)	600 mg BA 2 /day intravaginally for 14 days (*n* = 13)		
Guaschino et al. (24)	Prospective non-randomized study	22	RVVC	*C. albicans* (91%)	300 mg BA daily intravaginally for 14 days (*n* = 11)	1–6 months follow-up: mycological cure rates 88% (BA) and 85% (itraconazole)	
				*N. glabratus* SC (9%)	200 mg itraconazole PO daily for 3 days (*n* = 11)		
Marchaim et al. (25)	Retrospective cohort study	25	RVVC by fluconazole-resistant strains	*C. albicans*	Initial therapy: 600 mg BA 2 /day intravaginally for 14 days	Invariable mycologic eradication, successful treatment after maintenance	Maintenance success: higher fluconazole doses 11/11, ketoconazole 4/5, BA 3/5 and itraconazole 3/4
					Maintenance therapies with fluconazole PO 150–200 mg 2 /week (*n* = 11) or PO 100 mg/day (*n* = 1)		
					or BA intravaginally 3 /week (*n* = 5)		
					or ketoconazole PO 100 mg/day (*n* = 5)		
					or itraconazole PO 200 mg/day (*n* = 4)		
Powell et al. (26)	Retrospective observational study	67	Acute VVC	*N. glabratus* SC (63%)	600 mg BA daily intravaginally for 21–30 days (41 *N. glabratus* SC, 4 *C. parapsilosis* SC, 1 *P. kudriavzevii*)	BA: mycological cure rates 75% (*C. parapsilosis* SC), 78% (*N. glabratus* SC) and 100% (*P. kudriavzevii*)	
				*C. parapsilosis* SC (21%)	150–200 mg fluconazole PO daily (5 *N. glabratus* SC, 16 *C. parapsilosis* SC)	Fluconazole: mycological cure rates 60% (*N. glabratus* SC) and 81% (*C. parapsilosis* SC)	
				*P. kudriavzevii* (3%)			
				Other (11%)			
Sobel and Chaim (27)	Retrospective review	43	Uncomplicated/complicated VVC	*N. glabratus* SC	600 mg BA daily intravaginally for 14 days (*n* = 26)	BA: clinical improvement/cure 81%	
					100 mg clotrimazole daily intravaginally for 7 days (*n* = 11)	BA: mycological cure rate 77%	
					200 mg ketoconazole PO 2 /day for 5 days (*n* = 6)	Clotrimazole: clinical improvement/cure 46%	
						Clotrimazole: mycological cure rate 36%	
						Ketoconazole: clinical improvement/cure rate 50%	
						Ketoconazole: mycological cure rate 50%	
Sobel et al. (28)	Retrospective case series	111	VVC/RVVC	*N. glabratus* SC	600 mg BA daily intravaginally for 14–21 days	67% mycological cure rate	Duration of the treatment did nοt change the cure rates
Van Skyle et al. (29)	Double-blind comparative clinical trial	52	Acute VVC	*C. albicans*	600 mg BA daily intravaginally for 14 days	7–10 days follow-up: 92% mycological cure rate	
						30 days follow-up: 72% mycological cure rate	
Nyirjesy et al. (30)	Retrospective observational study	26	Chronic VVC	*C. parapsilosis* SC	600 mg BA 2 /day intravaginally for 14 days (*n* = 6)	1–4 months follow-up: 100% mycological cure rate (BA), 90% (fluconazole) and 100% (miconazole)	
					200 mg fluconazole PO 2 /week for 1 month (*n* = 19)		
					Miconazole one vaginal applicator once daily for 7 days (*n* = 1)		
File et al. (31)	Retrospective study	58	Fluconazole-resistant VVC	*C. albicans*	600 mg BA daily intravaginally for ≥ 14 days	7 days follow-up: mycological cure rate 85.7%, in subsequent follow-up: 80% mycological cure rate	14.3% mycological recurrence within 3 months
De Punzio et al. (32)	Double-blind, multicenter randomized study	70	Acute VVC	*C. albicans* (97%)	150 mg PO single dose of fluconazole (*n* = 38)	Day 7 follow-up: clinical cure rate 34% (fluconazole), 50% (itraconazole). Mycological cure rate 97% (fluconazole), 94% (itraconazole)	
				*N. glabratus* SC (1.5%)	200 mg itraconazole PO daily for 3 days (*n* = 32)	Day 21 follow-up: clinical cure rate 47% (fluconazole), 53% (itraconazole). Mycological cure rate 76% (fluconazole), 66% (itraconazole)	
				Other (1.5%)			
Fan et al. (33)	Open-label, randomized, parallel design study	290 (miconazole)	Severe VVC	*C. albicans* (89%)	1,200 mg miconazole intravaginally on days 1 and 4 (*C. albicans* 87%, *N. glabratus* SC 9%, *C. parapsilosis* SC 1%, *P. kudriavzevii* 2%, other 1%)	Day 14 follow-up: *C. albicans* mycological cure rates 80% (miconazole), 87% (fluconazole)	
		287 (fluconazole)		NAC spp. (11%)	150 mg fluconazole PO on days 1 and 4 (*C. albicans* 92%, *N. glabratus* SC 5%, *C. parapsilsosis* SC 1%, other 2%)	Day 14 follow-up: NAC spp. mycological cure rates 50% (miconazole) and 54% (fluconazole)	
						Day 35 follow-up: *C. albicans* mycological cure rates 68% (miconazole) and 71% (fluconazole)	
						Day 35 follow-up: NAC spp. mycological cure rates 45% (miconazole) and 54% (fluconazole)	
Ferahbas et al. (34)	Open-label, randomized, and comparative study	27	Acute VVC	Fluconazole: *C. albicans*	150 mg PO single dose of fluconazole (*n* = 16)	Fluconazole: 69% mycological cure rate	
				Itraconazole: 73% *C. albicans*, 27% *N. glabratus* SC	200 mg itraconazole PO daily for 7 days (*n* = 11)	Itraconazole: 50% mycological cure rate for *C. albicans*, 0% for *N. glabratus* SC	
Li et al. (35)	Prospective, randomized case-control study	66	Severe VVC	*C. albicans* (90%)	150 mg fluconazole PO on day 1 and 4	Day 7–14 follow-up: mycological cure rate 71%	
				*N. glabratus* SC (7%)		Day 28–35 follow-up: mycological cure rate 53%	
				*C. parapsilosis* SC (1%)			
				Other (2%)			
Mendling et al. (36)	Randomized, single-blind, parallel group, active-controlled, multi-center outpatient study	472	Acute VVC	Clotrimazole tablet: 96% *C. albicans*, 1% *N. glabratus* SC, 1% *P. kudriavzevii*, other 2%	500 mg single dose clotrimazole tablet intravaginally combined with 1% clotrimazole cream (*n* = 161)	Day 14 follow-up: clotrimazole tablet: 81% (*C. albicans*), 50% (*N. glabratus* SC), 50% (*P. kudriavzevii*) mycological cure rates	
				Clotrimazole cream: 94% *C. albicans*, 3% *N. glabratus* SC, 1% *P. kudriavzevii*, other 3%	10% clotrimazole single dose vaginal cream combined with 2% cream clotrimazole cream (*n* = 157)	Day 14 follow-up:clotrimazole cream: 76.4% (*C. albicans*), 20% (*N. glabratus* SC), 0% (*P. kudriavzevii*) mycological cure rates	
				Fluconazole: 96% *C. albicans*, 2% *N. glabratus* SC, 1% *P. kudriavzevii*, other 1%	150 mg PO single dose of fluconazole (*n* = 154)	Day 14 follow-up: fluconazole: 78% (*C. albicans*), 0% (*N. glabratus* SC), 0% (*P. kudriavzevii*) mycological cure rates	
						Day 28 follow-up: clotrimazole tablet: 81% (*C. albicans*), 0% (*N. glabratus* SC), 50% (*P. kudriavzevii*) mycological cure rates	
						Day 28 follow-up: clotrimazole cream: 78% (*C. albicans*), 20% (*N. glabratus* SC), 100% (*P. kudriavzevii*) mycological cure rates	
						Day 28 follow-up: fluconazole: 78% (*C. albicans*), 20% (*N. glabratus* SC), 100% (*P. kudriavzevii*) mycological cure rates	
O-Prasertsawat and Bourlert (37)	Single-blind, randomised, control trial	103	Acute VVC	Fluconazole (6/53 *N. glabratus* SC, 47/53 NA)	150 mg PO single dose of fluconazole (*n* = 53)	Mycological cure rate for *N. glabratus* SC: 17% (fluconazole), 50% (clotrimazole)	
				Clotrimazole (6/50 *N. glabratus* SC, 44/50 NA)	100 mg clotrimazole 2 /day intravaginally for 3 days (*n* = 50)		
Stein and Mummaw (38)	Randomized, double-blind, placebo-controlled, active-controlled trial	58	Acute VVC	*C. albicans* (98%)	200 mg clotrimazole daily intravaginally for 3 days (*n* = 20)	1 week follow-up: mycological cure rates 95% (clotrimazole), 73% (itraconazole)	
				NAC spp. (2%)	200 mg itraconazole PO daily for 3 days (*n* = 48)	4 weeks follow-up: mycological cure rates 83% (clotrimazole), 89% (itraconazole)	
Witt et al. (39)	Single-centre, prospective, randomised trial.	25	RVVC	*C. albicans*	Induction: 200 mg itraconazole PO 2 /day for a single day	12 months follow-up: 78% mycological cure rate	
					Maintenance: Itraconazole 200 mg PO 2 /day once a month		
Barnhart (40)	Multicenter, randomized, parallel-group, investigator-blind study	312	Acute VVC	*C. albicans* (90%)	1,200 mg intravaginally single dose of miconazole (daytime or bedtime treatment)	Mycological cure rates: 70% (daytime treatment), 64% (bedtime treatment)	
				*N. glabratus* SC (8%)			
				*C. parapsilosis* SC (1%)			
				*P. kudriavzevii* (1%)			
Wang et al. (41)	Multicenter, randomized, double-blind, phase three trial	159	Severe VVC	*C. albicans* (76%)	150 mg fluconazole PO on days 1 and 4	Day 14 follow-up: In all study group, 67% mycological cure, 50% clinical cure, 38% therapeutic cure	Clinical outcome for VVC caused by each of the NAC spp. was not available
				*N. glabratus* SC (17%)		Day 14 follow-up: In VVC caused by *C. albicans* alone, 79% mycological cure, 54% clinical cure, 46% therapeutic cure	
				*P. kudriavzevii* (1.5%)		Day 28 follow-up: In all studied group, 59% mycological cure, 56% clinical cure, 46% therapeutic cure	
				*C. parapsilosis* SC (1.5%)		Day 28 follow-up: In VVC caused by *C. albicans* alone, 71% mycological cure, 62% clinical cure, 56% therapeutic cure	
				Other (4%)			
Urünsak et al. (42)	Single-centre, randomized study	47	Acute VVC	*C. albicans* (77%)	A single-day treatment with 400 mg itraconazole PO	4 weeks follow-up: Clinical cure rates: 95% (*C. albicans*), 80% (*N. glabratus* SC) and 0% (*P. kudriavzevii*)	In short-term examination (1 week after treatment), mycological cure rates were ≤ 60% for each species
				*N. glabratus* SC (10%)			
				*P. kudriavzevii* (4%)			
				Other (9%)			
Furneri et al. (43)	Open-label, multicenter randomized controlled trial	90	Acute VVC	*C. albicans* (73%)	150 mg econazole daily intravaginally for 3 days	Mycological cure rates 84.8% (*C. albicans*), 40% (*N. glabratus* SC), 14.3% (*P. kudriavzevii*) and 0% (*C. parapsilosis* SC)	Only 2 cases of *C. parapsilosis* SC were involved in the trial
				*N. glabratus* SC (17%)			
				*P. kudriavzevii* (8%)			
				*C. parapsilosis* SC (2%)			
Sharma et al. (44)	Prospective, controlled trial	250	Acute VVC	*C. albicans*	400 mg ketoconazole PO for 5 days (*n* = 100)	1 month follow-up: mycological cure rate 80% (ketoconazole), 76% (miconazole) and 94% (combination of ketoconazole and miconazole)	Recurrence rate: 8-12% for each drug, 2% for the combination
					100 mg miconazole intravaginally daily for 14 days (*n* = 100)		
					Combination of the other two treatment regiments (*n* = 50)		
Lawrence et al. (45)	Open-label, randomized comparative clinical trial	153	Acute VVC	NA	600 mg single vaginal ovule of fenticonazole (*n* = 75)	7 days follow-up: mycological cure rates of 92% (fenticonazole) and 88.5% (clotrimazole)	
					500 mg single vaginal tablet of clotrimazole (*n* = 78)	1 month follow-up: mycological cure rates of 73.3% (fenticonazole) group and 66.7% (clotrimazole)	
Fan et al. (46)	Observational, prospective cohort	233	Uncomplicated/complicated VVC	*C. albicans* (92.5%)	400 mg miconazole daily intravaginally for 6 days	Day 14 follow-up: Mycological cure rates 94% (*C. albicans*) and 69% (*N. glabratus* SC)	
				*N. glabratus* SC (7%)		Day 35 follow-up: Mycological cure rates 86% (*C. albicans*), 63% (*N. glabratus* SC)	
				*C. parapsilosis* SC (0.5%)			
Brewster et al. (47)	Double-blind, randomized, clinical trial	51	Acute VVC	*C. albicans*	5 g of 2% fenticonazole cream applied daily for 7 days (*n* = 25)	Fenticonazole: 92.3% clinical cure	4–6 weeks after therapy: relapse rate 16% (fenticonazole) and 0% (clotrimazole)
					5 g of 1% clotrimazole cream applied daily for 7 days (*n* = 26)	Clotrimazole: 92% clinical cure	
Wiest et al. (48)	Investigator-blind, randomized, parallel-group, comparative trial	80	Acute VVC	*C. albicans*	600 mg fenticonazole intravaginally single dose (*n* = 40)	Day seven follow-up: mycological cure rates 88% (fenticonazole) and 93% (clotrimazole)	
					500 mg clotrimazole intravaginally single dose (*n* = 40)		
Nyirjesy et al. (49)	Open-label, multicenter, randomized, sponsor-blind, phase 2	19	Acute VVC	*C. albicans* (89.5%)	150 mg PO single dose of fluconazole	Day 28 follow-up: mycological cure rate 58% and clinical cure rate 53%	
				NAC spp. (10.5%)			
Bloch and Kretzel (50)	Open-label, randomized trial	109	Acute VVC	*C. albicans*	150 mg econazole daily intravaginally for 3 days (*n* = 55)	Day 21–32 follow-up: mycological cure rates 80% (3-day treatment) and 85.2% (14-day treatment). Clinical cure rates 85.4% and 98.2%, respectively	
					50 mg econazole daily intravaginally for 14 days (*n* = 54)		
Wiest and Ruffmann (51)	Open-label, randomized parallel group trial	60	Acute VVC	NA	200 mg fenticonazole intravaginally daily for 3 days (*n* = 20)	Mycological and clinical cure rates: 75-85%	No relapses noted 2 weeks after the end of the treatment
					600 mg fenticonazole single dose intravaginally (*n* = 20)	Day seven follow-up: mycological cure rates 80% (200 mg), 75% (600 mg), 85% (1,000 mg)	
					Fenticonazole 1000 mg intravaginally single dose (*n* = 20)		
Murina et al. (52)	Prospective study	80	Acute VVC	NA	150 mg fluconazole PO two doses 3 days apart (*n* = 40)	7 days follow-up: clinical cure rate 77.5% (fluconazole), 80% (fenticonazole)	Symptoms relieved in lower time in fenticonazole (2.3 days vs. 4.5 days, respectively)
					600 mg fenticonazole intravaginally two doses 3 days apart (*n* = 40)		
Sobel et al. (53)	Open-label, randomized, multicenter study	151	RVVC	NA	100 mg clotrimazole intravaginally daily for 7 days (*n* = 77)	1 month follow-up: clotrimazole: 82% therapeutic cure rate, ketoconazole: 80% therapeutic cure rate	At the follow-up therapeutic failure rates were 63% (clotrimazole) and 53% (ketoconazole)
					400 mg ketoconazole PO daily for 17 days (*n* = 74)		
Friese et al. (54)	Prospective, multicenter, randomized, case-control study	247	Acute VVC	*C. albicans* (72%)	100 mg clotrimazole intravaginally daily for 6 days	87% mycological cure rate among all species	For *N. glabratus* SC alone therapeutic cure rate was lower than 59%
				*N. glabratus* SC (15%)			
				Other (13%)			
Hughes and Kriedman (55)	Randomized, double-blind, placebo-controlled trial	10	Acute VVC	*C. albicans*	A single dose of 500 mg clotrimazole intravaginally	1 month follow-up: mycological and clinical cure rate 90%	
Bro (56)	Randomized, double-blind, placebo-controlled trial	55	Acute VVC	*C. albicans* (65%)	A single dose of 500 mg clotrimazole intravaginally	7–10 days follow-up: mycological cure rate 62%	
				NAC spp. (35%)			
Fernández-Alba et al. (57)	Open-label, prospective, multicenter pilot study	29	Acute VVC	*C. albicans*	1 g fenticonazole intravaginally on days 1 and 3	Day eight follow-up: mycological cure rate 90%	No relapse according to 28 day follow-up
Reyes et al. (58)	Randomized controlled trial	80	Acute VVC	Fluconazole: 93% *C. albicans*, 7% other	200 mg fenticonazole daily intravaginally for 3 days (*n* = 40)	Mycological cure rate 97.5% for both treatments	
				Miconazole: 85% *C. albicans*, 15% other	400 mg miconazole daily intravaginally for 3 days (*n* = 40)		
Dellenbach et al. (59)	Multicenter, randomized double-blind study	160	Acute VVC	*C. albicans* (97%)	A single 150 mg econazole 1 /week (one or two administrations until clinical cure is achieved)	Mycological cure rates after one or two administrations: 56-59%	
				*N. glabratus* SC (1%)			
				Mixed (two different *Candida* spp., 2%)			
Puolakka and Tuimala (60)	Open-label randomized controlled trial	140	Acute VVC	NA	400 mg ketoconazole PO daily for 3 days (*n* = 49)	1 week follow-up: mycological cure rates 67% (400 mg ketoconazole), 78% (200 mg ketoconazole) and 81% (miconazole)	
					200 mg ketoconazole PO daily for 6 days (*n* = 42)		
					100 mg miconazole daily intravaginally for 14 days (*n* = 49)		
Goswami et al. (61)		114	Diabetic/non-diabetic women with VVC	*C. albicans* (32%)	150 mg PO single dose of fluconazole	Mycological cure rates: 64% (*C. albicans*), 19% (*N. glabratus* SC), 75% (*C. parapsilosis* SC), 50% (*P. kudriavzevii)*	
				*N. glabratus* SC (51%)			
				*C. parapsilosis* SC (4%)			
				*P. kudriavzevii* (1%)			
				Other (12%)			
Mollazadeh-Narestan et al. (62)	Triple-blinded randomized, controlled Trial	40	Acute VVC	Out of 40 patients: *C. albicans* (55%)	150 mg PO single dose of fluconazole	35–40 and 60–65 days follow up: mycological cure rates 73% (*C. albicans*), 29% (*N. glabratus* SC), 80% (*C. parapsilosis* SC), 0% (*P. kudriavzevii*)	7/40 individuals were lost to follow-up, data on the species they had were unavailable.
				*N. glabratus* SC (17.5%)			
				*C. parapsilosis* SC (12.5%)			
				*P. kudriavzevii* (15%)			

BA, boric acid; NA, not available; NAC, non-*albicans Candida;* PO, per os; RVVC, recurrent vulvovaginal candidiasis; SC, species complex; VVC, vulvovaginal candidiasis.

For *N. glabratus* species complex (SC) VVC mycological cure rates were 0%–28.6% with a single dose of 150 mg fluconazole (PO), 40% with 150 mg econazole (intravaginally) daily for 3 days, 0%–50% with 100 mg clotrimazole (intravaginally) once or twice a day for 7 and 3 days, respectively, or with 500 mg clotrimazole single dose accompanied with 1% clotrimazole vaginal cream or with 10% clotrimazole single dose cream in combination with 2% clotrimazole cream, 150 mg daily for 3 days econazole or clotrimazole intravaginally, 63%–69% with 400 mg miconazole (intravaginally) daily for 6 days, 50% with 200 mg ketoconazole PO twice a day for 5 days, 0%–50% with 200 mg itraconazole PO for 7 or 14 days but 80% with 400 mg itraconazole PO single day treatment, 63.6%–85% with 600 mg boric acid intravaginally daily for 14 days or longer (21–30 days). There are no clinical data for fenticonazole and *N. glabratus* SC.

For *C. parapilosis* SC mycological cure rates were 81%–90% with 150–200 mg fluconazole PO twice a week for 1 month, 100% with one vaginal applicator of miconazole for 7 days, 75%–100% with 600 mg boric acid twice a day for 14 days or once a day for 21–30 days and 0% with 150 mg/L econazole intravaginally (this study included only two cases of *C. parapsilosis* SC VVC).

#### Our data set

**Fig 1:**
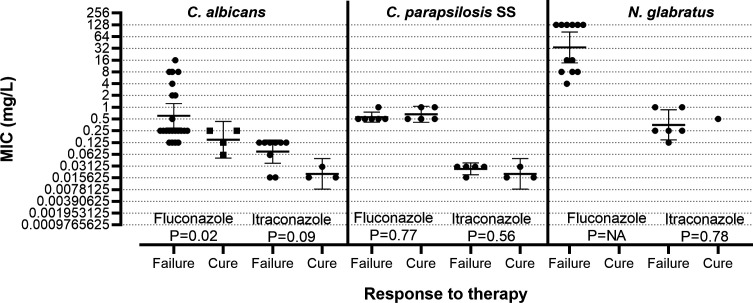
Comparison of fluconazole and itraconazole MICs between VVC cases with failure and cure after oral antifungal therapy for *C. albicans*, *C. parapsilosis* SS, and *N. glabratus*. Error bars indicate geometric mean and 95% confidence interval. ANOVA *P* values of Sidak’s multiple comparison test are shown.

Among patients with isolates tested in the present study, we identified 56 VVC patients treated with fluconazole, 7 with the topical formulation 0.5% w/w gel (3 with *C. albicans,* 2 with *C. parapsilosis* SS, and 2 with *N. glabratus)* and 49 with the oral dose of 150 mg once (26 with *C. albicans*, 11 with *C. parapsilosis* SS, and 12 with *N. glabratus*) and 26 patients treated with oral itraconazole of 200 mg/day for 3 days (12 with *C. albicans*, 8 *C. parapsilosis* SS, and 6 with *N. glabratus)*. Therapeutic failures were found for all patients treated with a topical formulation of fluconazole. For the orally administered drugs, the MICs and clinical response for each species and drugs are shown in Fig. 1. Statistically significant MIC differences (*P* = 0.02) were found for *C. albicans* and fluconazole between cases with therapeutic failure and cure with GM MIC (range) 0.60 (0.125–16) vs 0.15 (0.06–0.25) mg/L and 32% vs 0% of isolates having MICs ≥ 0.5 mg/L, respectively. Bordering significant differences (*P* = 0.09) were found for *C. albicans* and itraconazole between cases with therapeutic failure and cure with GM MIC 0.07 (0.016–0.125) vs 0.02 (0.016–0.03) mg/L, respectively, and 78% vs 0% of isolates, respectively, having MICs ≥ 0.06 mg/L. No differences were found for the other species.

## DISCUSSION

The MIC distributions of oral and topical antifungal drugs used for VVC treatment were analyzed and local ECOFFs were determined for the EUCAST methodology visually and statistically using the ECOFFinder and derivatization. Consensus ECOFFs were compared with fluconazole susceptibility data as a significant correlation was found between the MICs of fluconazole and other azoles. Fluconazole-resistant isolates were also itraconazole-resistant, supporting further the cross-resistance hypothesis which was utilized in the present study to determine local ECOFFs for other azoles. More non-WT isolates were found for *N. glabratus* (10%–35% for all azoles except miconazole and econazole) followed by *C. albicans* (15%–16% for fluconazole, ketoconazole, miconazole, and econazole, 5%–8% for itraconazole, clotrimazole, and fenticonazole) and *C. parapsilosis* SS (≤ 3% for all azoles). Azoles’ MICs of fluconazole-susceptible isolates were higher than the local ECOFFs for all drugs except itraconazole and clotrimazole for *C. albicans* indicating that some non-WT isolates may be treated with these two drugs providing sufficient exposure is attained. The opposite was found for *N. glabratus* for all azoles except itraconazole and clotrimazole indicating that exposure may not be enough to treat *N. glabratus* infections with most azoles whereas itraconazole and clotrimazole may have some role in *N. glabratus* VVC. Boric acid had a uniform *in vitro* activity with narrow MICs that were not correlated with azole MICs.

Clinical data also support the estimated local ECOFFs. Based on our review, the mycological cure was usually > 60% for *C. albicans* and *C. parapsilosis* SC for azoles and boric acid indicating good clinical efficacy. However, for *N. glabratus* SC mycological cure was < 50% for most azoles except miconazole (63%–69%) and itraconazole (80% with the 400 mg dose), and 85% for boric acid indicating that *N. glabratus* SC should be considered intrinsically resistant to most azoles except itraconazole and perhaps miconazole for which dose optimization strategies could increase clinical efficacy. Regarding our clinical data for women treated with fluconazole and itraconazole, *C. albicans* VVC outcome showed significantly different MICs, almost all women with *N. glabratus* VVC failed to be treated with both azoles and MICs of *C. parapsilosis* SS failed to be correlated with treatment outcome.

When examining our MIC data, while no official quality control MIC data are available for most drugs tested, the MICs of *C. parapsilosis* ATCC 22019 and *C. krusei* ATCC 6258 strains were within the EUCAST target values for fluconazole and itraconazole and, for the rest of the drugs, they were consistent with studies using EUCAST or CLSI methods (Table 1). Regarding the clinical isolates, our data align with existing MIC distributions where EUCAST methodology was used for fluconazole, itraconazole, miconazole, and clotrimazole for *C. albicans*, *C. parapsilosis* SC, and *N. glabratus* SC (±1 twofold dilution differences of modal MICs) (16, 18, 63, 64). Comparing to CLSI MIC data, our modal MICs were the same for ketoconazole and econazole for the three species but 2–3 twofold dilutions lower for miconazole, clotrimazole, itraconazole, and econazole for *C. parapsilosis* SC and for clotrimazole and *C. albicans* (65). However, lower MICs for the latter drug-species pair have been also reported (66). Similarly, *P. kudriavzevii* MICs for fluconazole, ketoconazole, miconazole, and econazole agree with previous CLSI MIC data, while itraconazole MICs were lower in our study (MIC_50_ 0.25 mg/L vs 0.06 mg/L) (65). Fenticonazole MICs for *C. albicans* and *N. glabratus* SC align with previous studies with the CLSI method (67, 68) although 2 twofold lower MICs than our MICs have been reported (19, 69). Boric acid MIC data for *Candida* species are extremely limited, but quality control MICs align with previous CLSI MICs for ATCC 22019 and ATCC 6258 strains (20) and the MICs of 46 fluconazole-susceptible *C. albicans* were ≤ 2,500 mg/L (70) in agreement with the MIC data of clinical isolates of the present study.

Fluconazole is commonly used for acute VVC treatment, prophylaxis, and RVVC maintenance (71). However, its long-term efficacy is debatable, and overexposure may lead to resistance (71). For acute *C. albicans* VVC, itraconazole and clotrimazole are promising alternatives, as both demonstrated similar *in vitro* activity (5%–8% non-WT) and were more effective than fluconazole (16% non-WT). These *in vitro* findings have clinical relevance in studies summarized in Table 6, as mycological cure rates > 69% were reported in *C. albicans* VVC cases treated with azoles. Specifically, in a comparative study on acute VVC, three treatments were evaluated: topical clotrimazole (500 mg vaginal pessary and 1% cream), oral itraconazole (200 mg twice daily for 1 day), and fluconazole (a single 150 mg dose) (72). Mycological cure rates of 95%, 96%, and 83% for clotrimazole, itraconazole, and fluconazole, respectively (*P* = 0.008), were recorded. Similarly, the proportion of patients achieving clinical cure showed a consistent pattern across treatments (itraconazole 80%, clotrimazole 80%, fluconazole 62%) (72).

VVC caused by *N. glabratus* is challenging to treat, mainly because it possesses resistance mechanisms to azoles. Investigating the role of azoles in treating *N. glabratus* VVC, both fluconazole and fenticonazole displayed better *in vitro* activity against *C. albicans* compared to *N. glabratus* (16% vs 23% non-WT to fluconazole and 5% vs 10% non-WT to fenticonazole, respectively). Indeed women treated with a single-day regimen of oral fluconazole combined with topical fenticonazole showed 95.5% effectiveness against *C. albicans* and 83.3% against *N. glabratus* SC (73). Consistent with these high rates of clinical cure, among all azoles in our study, fenticonazole had the lowest percentage of non-WT phenotypes for both *C. albicans* and *N. glabratus* indicating that fenticonazole could be a promising agent in managing VVC including some fluconazole-resistant isolates. For fluconazole-susceptible increased exposure *N. glabratus* isolates, the local ECOFFs were higher than the MIC cut-offs for azoles indicating poor activity, except itraconazole and clotrimazole that had the same local ECOFFs suggesting a role of these azoles in treating *N. glabratus* VVC. Indeed, as summarized in Table 6 the mycological cure of patients with acute VVC by *N. glabratus* SC is usually <59% for different azoles with higher rates reported for 400 mg miconazole intravaginally for 6 days (69%), and a single-day treatment with 400 mg itraconazole PO (80%) (42). These findings show poor efficacy of azoles against *N. glabratus* SC VVC except itraconazole. Notably, no cure was found for 2 *P. kudriavzevii* cases supporting our findings that most *P. kudriavzevii* isolates had MICs higher than *C. albicans* ECOFFs including itraconazole and may be resistant (42).

Delving deeper into our investigation of NAC species, *C. parapsilosis* SS showed higher MICs compared to *C. albicans*, as found previously (74, 75). Existing literature commonly associates VVC attributed to *C. parapsilosis* SC with low recurrence rates and satisfactory response to treatment with topical azole preparations (30, 76). This is in line with the low percentage of non-WT phenotypes found in the present study based on the estimated local ECOFFs. Although the role of *C. parapsilosis* SC as a vaginal pathogen is uncertain, mycological cure rates were >90% with 200 mg fluconazole PO twice weekly for 1 month and 100% with miconazole intravaginally once daily for 7 days (30).

Taking into account the diverse responses of different *Candida* species to azoles, it becomes evident that establishing ECOFFs is crucial for effectively managing VVC. Other MIC cut-offs have been previously used, like the ≥ 64 mg/L for fluconazole and ≥ 1 mg/L for miconazole, ketoconazole, and itraconazole to detect resistance in a study involving 89 strains from VVC (77). Although these endpoints were not species-specific and were employed to interpret results obtained using the CLSI method among *C. albicans* and NAC isolates, they align with the local ECOFFs determined in the present study for miconazole, ketoconazole, itraconazole, and *N. glabratus* (77). Other interpretive MIC cut-offs recently used to assess the resistance of VVC pathogens for miconazole and clotrimazole were ≥ 16 mg/L and ≥ 1 mg/L, respectively (78). These criteria would underestimate the resistance for species for which the MIC distribution of WT isolates is low like *C. albicans*. As MIC distributions are different among the species tested, so are ECOFFs.

The percentage of non-WT phenotypes among azoles is similar for each species reflecting not only the importance of species identification and susceptibility testing for appropriate VVC treatment, but also the cross-resistance phenomenon further supporting the local ECOFFs determined in this study. Cross-resistance in *Candida* typically arises through mutations in the sterol 14-demethylase enzyme (ERG11), overexpression of drug efflux pumps, upregulation of the ERG11 gene, and alterations in ergosterol biosynthesis (79). Intriguingly, approximately 33% of amino acid substitutions in the ERG11 of *C. albicans* lead to resistance, and among these, a high percentage (88%) confers cross-resistance to multiple azoles (80).

In contrast, boric acid was the only antifungal under study whose *in vitro* activity remained unaffected by the pathogen’s susceptibility to fluconazole for all species tested. Unlike azoles, boric acid affects different biological process of fungi like mitochondrial enzymes, hyphal growth and biofilm formation. A recent study about VVC revealed a negative correlation between resistance to fluconazole and boric acid MICs in *C. albicans* or no correlation in *N. glabratus* SC and *C. parapsilosis* SC from disk assays (81). Boric acid has gained attention as an alternative treatment against VVC by azole-resistant strains, with high rates of clinical cure for *N. glabratus* SC and *P. kudriavzevii* (82, 83). We estimated a higher *in vitro* efficacy of boric acid for *P. kudriavzevii* (GM MIC 800 mg/L) compared to other species, and we also observed no non-WT strains for all species according to the determined ECOFFs. Similarly, out of 165 *C. albicans*, 50 *N. glabratus* SC, and 20 *C. parapsilosis* SC, only one *C. parapsilosis* SC strain exhibited a considerably higher MIC for boric acid (81). Clinically, boric acid has demonstrated a significantly higher cure rate (72% compared to 33% for fluconazole) among patients with acute *N. glabratus* SC VVC, when they were treated with 600 mg of boric acid daily for 14 days or a 150 mg single dose of fluconazole (21).

To determine clinical breakpoints for drugs used for VVC, MIC-clinical outcome data are needed, but such studies remain extremely limited. Although a correlation between the MIC and therapeutic response to oral fluconazole has been demonstrated for oropharyngeal candidiasis (84), *in vitro* susceptibility was poorly correlated with the clinical response to oral fluconazole (single or two 150 mg doses) for *C. albicans* VVC. However, a higher mycological response was found for isolates with CLSI MICs ≤ 1 mg/L compared to isolates with MICs > 1 mg/L (80% vs 67% on day 14 and 61% vs 50% on day 31) (85). It’s alarming that the clinical success in the same study was high for isolates with MICs ≥ 64 mg/L (100% on day 14 and 82% on day 34) indicating that despite the clinical response women remained colonized and may be at risk for recurrence of symptomatic disease (85). Azoles because of their fungistatic action may reduce fungal burden in vaginal fluid alleviating clinical symptoms without eradicating fungi, at 1× MIC azoles reduce *Candida* conidia by 1 log compared to drug-free control (86). Considering the mean (range) peak vaginal concentrations of fluconazole of 2.42 (1.10-3.90) mg/L 8–24 h after a single oral 150 mg dose (87), such an effect can be observed for WT *C. albicans* but not for WT *N. glabratus* isolates.

Our data set indicates that topical therapy with fluconazole is not effective even for isolates with low MICs. For standard doses of oral fluconazole and itraconazole, the therapeutic cure was associated with *C. albicans* isolates with MICs lower than local ECOFFs (0.5 mg/L and 0.06 mg/L, respectively). High failure rates were found for *N. glabratus* indicating that azoles are not active against this species whereas no correlation between MIC and clinical response was found for *C. parapsilosis* SS questioning the pathogenicity of this species in VVC. From a PK/PD perspective, based on the 24 h average vaginal concentration of itraconazole ~0.25 µg/g in vaginal tissue and 30% interindividual variation in plasma levels observed with the 200 mg PO dose (88, 89) vaginal concentrations may vary between 0.1-0.4 µg/g. Based on the universal PK/PD target for azoles and *Candida* species of 25 fAUC/MIC (90), the latter target can be attained for isolates with MIC up to 0.06 mg/L supporting a conservative breakpoint of 0.06 mg/L. Similarly for fluconazole, vaginal concentration of 2.42 µg/g was reported after 150 mg PO dose (87) which may vary between 0.8 and 4 µg/g considering an interindividual variation of 30%. This can support a conservative breakpoint at the local ECOFF of 0.5 mg/L determined in the present study. A 10–100-fold interindividual variation was found in miconazole vaginal concentrations after a 100 mg intravaginal dose (1.5–14.5 mg/L at C_max_ and 0.2–14.4 mg/L at 24 h) which may explain the wide variation in efficacy (91). Although drug concentrations of topical agents are high in the vaginal secretions, their concentration in the vaginal epithelium, the site of infection, is unknown. Low penetration in vaginal epithelium may explain the better efficacy of oral therapy compared to topical. Furthermore, *Candida* may form biofilm in the vaginal epithelium against which azoles do not have activity (92). Several factors may influence the efficacy of antifungal drugs hindering any effect of MIC on clinical outcomes (71). More pharmacokinetic and pharmacodynamic studies of vaginitis are required in order to understand the PK/PD characteristics of both topical and oral agents against the different species.

In conclusion, in an attempt to fill the gap in *in vitro* susceptibility testing of drugs used for VVC, local ECOFFs have been determined for three major *Candida* species using modern techniques and utilizing fluconazole susceptibility and clinical data. The ECOFFs estimated in this study derived from a certain number of Greek isolates. Therefore, there is a continued need to establish official ECOFFs according to EUCAST guidelines, which require data from multiple centers (at least five) and for many isolates (at least 15 per species). Until then, researchers may use current ECOFFs providing that the MIC distributions they generate are similar to the MIC distributions of the present study (same modal MIC). Treatment for non-complicated *C. albicans* VVC should focus on optimizing current azole regimens. The pathogenicity of *C. parapsilosis* SS requires further exploration, while *N. glabratus* VVC may be better managed with the concurrent use of topical and oral azoles or non-azole antifungals, depending on the isolate’s susceptibility. However, it is important to note the absence of official quality control ranges for most VVC drugs necessitating reliance on data published by other researchers who employed the EUCAST methodology. We also had to depend on official EUCAST CBPs to investigate MIC endpoints of other azoles that predict fluconazole resistance. Previous studies using the CLSI methodology with an acidic pH medium (reflecting the vaginal environment) found elevated fluconazole MICs (93). Therefore, official CBPs may not fully apply to VVC, as both CLSI and EUCAST methodologies are standardized at pH 7.0, and further research on the impact of pH in AFST including clinical data would enlighten this issue. Future efforts should also aim to incorporate these findings into clinical decision-making, optimizing the treatment of VVC and helping to prevent resistance development.
